# CoolMPS for robust sequencing of single-nuclear RNAs captured by droplet-based method

**DOI:** 10.1093/nar/gkaa1127

**Published:** 2020-12-02

**Authors:** Oliver Hahn, Tobias Fehlmann, Hui Zhang, Christy N Munson, Ryan T Vest, Adam Borcherding, Sophie Liu, Christian Villarosa, Snezana Drmanac, Rade Drmanac, Andreas Keller, Tony Wyss-Coray

**Affiliations:** Department of Neurology and Neurological Sciences, Stanford University School of Medicine, Stanford, CA, USA; Wu Tsai Neurosciences Institute, Stanford University School of Medicine, Stanford, CA, USA; Chair for Clinical Bioinformatics, Saarland University, Saarbrücken, Germany; Center for Bioinformatics, Saarland Informatics Campus, Saarbrücken, Germany; Department of Neurology and Neurological Sciences, Stanford University School of Medicine, Stanford, CA, USA; Department of Neurology and Neurological Sciences, Stanford University School of Medicine, Stanford, CA, USA; Department of Neurology and Neurological Sciences, Stanford University School of Medicine, Stanford, CA, USA; Department of Chemical Engineering, Stanford University, Stanford, CA, USA; MGI, 2904 Orchard Pkwy, San Jose, CA, USA; MGI, 2904 Orchard Pkwy, San Jose, CA, USA; MGI, 2904 Orchard Pkwy, San Jose, CA, USA; MGI, 2904 Orchard Pkwy, San Jose, CA, USA; MGI, 2904 Orchard Pkwy, San Jose, CA, USA; Department of Neurology and Neurological Sciences, Stanford University School of Medicine, Stanford, CA, USA; Chair for Clinical Bioinformatics, Saarland University, Saarbrücken, Germany; Center for Bioinformatics, Saarland Informatics Campus, Saarbrücken, Germany; Department of Neurology and Neurological Sciences, Stanford University School of Medicine, Stanford, CA, USA; Wu Tsai Neurosciences Institute, Stanford University School of Medicine, Stanford, CA, USA; Department of Bioengineering, Stanford University, Stanford, CA, USA; Paul F. Glenn Center for the Biology of Aging, Stanford University School of Medicine, Stanford, CA, USA

## Abstract

Massively-parallel single-cell and single-nucleus RNA sequencing (scRNA-seq, snRNA-seq) requires extensive sequencing to achieve proper per-cell coverage, making sequencing resources and availability of sequencers critical factors for conducting deep transcriptional profiling. CoolMPS is a novel sequencing-by-synthesis approach that relies on nucleotide labeling by re-usable antibodies, but whether it is applicable to snRNA-seq has not been tested. Here, we use a low-cost and off-the-shelf protocol to chemically convert libraries generated with the widely-used Chromium 10X technology to be sequenceable with CoolMPS technology. To assess the quality and performance of converted libraries sequenced with CoolMPS, we generated a snRNA-seq dataset from the hippocampus of young and old mice. Native libraries were sequenced on an Illumina Novaseq and libraries that were converted to be compatible with CoolMPS were sequenced on a DNBSEQ-400RS. CoolMPS-derived data faithfully replicated key characteristics of the native library dataset, including correct estimation of ambient RNA-contamination, detection of captured cells, cell clustering results, spatial marker gene expression, inter- and intra-replicate differences and gene expression changes during aging. In conclusion, our results show that CoolMPS provides a viable alternative to standard sequencing of RNA from droplet-based libraries.

## INTRODUCTION

Next generation sequencing has accelerated biomedical research and diagnostics alike, particularly the sequence-by-synthesis approach (1–3). Applications of this technology for sequencing cDNA libraries generated from RNA molecules (RNA-seq) have seen tremendous advancements and yielded a wide range of applications that are continuously expanded (3). Illumina sequencers are the most frequently used instruments, and several commercial and/or customized RNA-seq methods generate exclusively Illumina-compatible sequencing libraries (3). These sequencers rely on PCR-based clonal amplification of cDNA fragments and use dye-labeled nucleotides to monitor the synthesis reaction. Interestingly, an alternative method, called CoolMPS, has been recently commercialized by BGI (4). This method uses circularized libraries that are amplified in a rolling-circle, and therefore, PCR-free, manner. The results are ‘DNA nanoballs’, that are then sequenced through application of four fluorescently labeled antibodies. These antibodies recognize and distinguish the four bases A, T, C, G, thereby allowing sequence-by-synthesis with label-free nucleotides (4). Given its distinct chemistry, sequencing libraries must either be generated with CoolMPS-compatible reagents or Illumina-compatible libraries must be chemically converted to induce circularization.

Single-cell sequencing technologies have significantly impacted life sciences and permitted genetic, epigenetic and transcriptomic profiling at previously unprecedented resolution (5). Single-cell and single-nucleus RNA-sequencing (scRNA-seq, snRNA-seq) in combination with droplet-based technologies such as the Chromium 10X platform (6) are among the most widely used approaches (5). These assays have been applied in efforts to map the transcriptomes of all cell types of whole organisms (5,7,8), as well as identification of changes in cell composition and gene expression under perturbations such as disease and aging (7,9–11). Previous studies have demonstrated that single-cell libraries derived via the Smart-seq2 and Chromium 10X (version 2 chemistry) protocol can be converted and sequenced using the conventional MGISEQ technology (12,13). However, the performance of DNA nanoball-conversion and subsequent sequencing of single-libraries with CoolMPS has not been assessed yet. Additionally, the Chromium 10X version 2 chemistry has been recently discontinued and updated significantly (to so-called ‘version 3′), and the changes allow for far more complex libraries yielding almost twice the number of detectable genes and transcripts per cell (14). Finally, conversion assessments have been limited to live cells in suspension. snRNA-seq from frozen tissues holds the promise of sequencing critical tissues that either cannot be easily dissociated or were biobanked with a protocol that does not allow for single-cell dissociation after thawing (15). This excludes a wide range of relevant tissues, many of them from human patients.

The brain, arguably the most complex tissue in mammalian anatomy, is herein a prime example of how the granularity of droplet-based single-cell assays has significantly contributed to our understanding of its plasticity and function in aged individuals and in age-related diseases (9,16–21). One region of interest in the aging brain is the hippocampus, which is generally considered as a key center for learning and short-term memory formation, and both functions are known to decline with age (22). The hippocampus consists of diverse canonical cell types at varying abundance, such as neurons, oligodendrocytes and astrocytes, which undergo distinct and common transcriptional shifts during aging (19). Furthermore, each cell type can have multiple sub-populations of different anatomical origin or functional roles, e.g. the pyramidal neurons of the trisynaptic loop (CA1, CA2, CA3) and the granule neurons of the dentate gyrus (DG) (23).

In this study, we assessed the performance of Chromium 10X snRNA-seq libraries that were chemically converted to be compatible with and sequenced using CoolMPS. Using hippocampi of young and old mice as input tissue, we comparatively analyse data generated from native and CoolMPS-compatible libraries (sequenced on an Illumina and BGI sequencer, respectively) across a range of parameters, such as overall read distribution, barcode recovery, detection of contaminants, cell clustering, cell type identification and differential expression. We report remarkably consistent performance between native and converted libraries. Thus, we consider sequencing droplet-based single-cell RNA-seq libraries that are converted to be compatible with the CoolMPS method to be a viable alternative to the current standard.

## MATERIALS AND METHODS

### Animals

Old C57BL/6J male wild-type mice (18 months) were obtained from the National Institute on Aging, and young C57BL/6J males (2 months) were purchased from The Jackson Laboratory. Throughout the whole manuscript we refer to these samples—and the libraries generated from their tissues—as ‘Y1’, ‘Y2’ (young males replicate 1 and 2, respectively), ‘O1’ and ‘O2’ (old males replicate 1 and 2, respectively). Mice were housed under a 12-h light–dark cycle in pathogen-free conditions in accordance with the Guide for Care and Use of Laboratory Animals of the National Institutes of Health.

All animal procedures were approved by the VA Palo Alto Committee on Animal Research and the institutional administrative panel of laboratory animal care at Stanford University. Male mice were used for all experiments.

### Nuclei isolation and Chromium 10X library generation

Nuclei were isolated as described previously in (15). Per replicate, we isolated nuclei from the flash-frozen, whole hippocampus of the right hemisphere. All reagents were placed on ice. Frozen tissue was placed on a lab dish on ice and covered with 1 ml lysis buffer from the Nuclei EZ Prep Kit (Sigma-Aldrich, St. Louis, USA). Tissue was cut into 2 mm-small pieces and the tissue-buffer mix was transferred on a 2 ml tissue grinder placed on ice. 1 ml of fresh lysis buffer was used to rinse the lab dish and then loaded on the tissue grinder as well (2 ml total volume). Tissues were homogenized with 25 strokes with pastel A followed by 15 strokes with pastel B. Tissue homogenate was transferred to a fresh tube on ice. The tissue grinder was rinsed with 2 ml of fresh lysis buffer, and then transferred to the same tube holding the homogenate (4 ml total). The tube was incubated on ice for 5 min. Nuclei were pelleted with 5 min centrifugation at 500 × g, and then the pellet was resuspended with 4 ml fresh lysis buffer. Following one more 5 min centrifugation at 500 × g, lysis buffer was removed, and the pellet was washed with 4 ml PBS. Nuclei were pelleted and resuspended in 500 μl PBS containing 2 U/ml Protector RNase Inhibitor (Sigma-Aldrich, St. Louis, USA). Nuclei solution was parsed through a 40 μm mesh. Nuclei were counted and concentrations adjusted to ∼1 mio nuclei/ml.

Reagents of the Chromium Single Cell 3′ Library & Gel Bead Kit v3 (10X Genomics, Pleasanton, USA) were thawed and prepared according to the manufacturer's protocol. Nuclei/master mix solution was adjusted to target 7,500 nuclei per sample and loaded on a standard Chromium Controller (10X Genomics, Pleasanton, USA), according to the manufacturer's protocol. All reaction and quality control steps, including library construction (using Chromium Single Cell 3′ Library Construction Kit v3) were conducted according to the manufacturer's protocol and with recommended reagents, consumables and instruments. Quality control of cDNA and libraries was conducted using a Bioanalyzer (Agilent, Santa Clara USA) at the Stanford Protein and Nucleic Acid Facility (http://pan.stanford.edu/index.html).

### Illumina sequencing

Illumina sequencing of 10X snRNA-seq libraries was performed by Novogene Co. Inc. (Sacramento, USA; https://en.novogene.com/). Multiplexed libraries were sequenced with 2 × 150-bp paired-end (PE) reads in a single S4 lane on a Illumina Novaseq S4 (Illumina, San Diego, USA), targeting 215 million reads per library. Base calling, demultiplexing and generation of FastQ files was conducted by Novogene.

snRNA-seq data for the native and converted libraries are available under GEO ID GSE150284 (https://www.ncbi.nlm.nih.gov/geo/query/acc.cgi?acc=GSE150284).

### Library conversion and CoolMPS sequencing

Libraries generated using the Chromium 10X Single Cell 3′ (version 3) kit require a conversion step using the MGIEasy Universal Library Conversion kit (Part Number: 1000004155, MGI Tech Co., Ltd, Shenzhen, China) before DNA nanoballs (DNBs) can be generated. For each library, 50 ng of final 10X library product was amplified using 5 cycles of PCR to incorporate a 5′ phosphorylation on the forward strand only. Purified PCR product was then denatured and mixed with a ‘splint’ oligonucleotide that is homologous to both P5 and P7 adapter regions of the library to generate a circle. A ligase reaction was then performed to create a complete ssDNA circle of the forward strand. Then an exonuclease digest was performed to remove single stranded non-circularized DNA molecules and splint oligonucleotide. DNBs were then prepared using the DNBSEQ-G400RS High-throughput Sequencing Set (FCL PE100—Part Number 1000016950, MGI Tech Co., Ltd, Shenzhen, China), according to the manufacturer's protocol https://en.mgitech.cn/download/files/hao_id/2/p/2. Rolling circle replication time for DNB preparation was set to 25 min. DNBs were loaded onto DNBSEQ-G400 4-lane flowcell provided with the PE100 sequencing set by manual pipetting of DNBs mixed with loading buffer into flowcell lanes using the MGIDL-200H DNB loader as described in the CoolMPS High-throughput Sequencing Set User Manual provided with the kit. Sequencing primers in the MGI CoolMPS PE100 kit were replaced with the primers provided in the High-Throughput Pair-End Sequencing Primer Kit (App-A) (Part Number 1000004156, MGI Tech Co., Ltd, Shenzhen, China) according to the instructions described in the Primer Kit manual. Sequencing was performed on a DNBSEQ-G400 (MGI Tech) using a customized program with 28 sequencing cycles (cell barcode) for the 1st strand and 98 cycles of cDNA followed by eight cycles of the sample barcode reading for the second strand. Whereas the standard sequencing-by-synthesis approach relies on incorporation of labeled nucleotides, the CoolMPS method sequences via the incorporation of unlabeled, reversibly terminated nucleotides. The fluorescent signal to detect the incorporated bases is generated by using base-specific 3′ block-dependent fluorescently labeled antibodies. After each cycle, the bound antibodies are removed and 3′ blocking moiety on the sugar group of the nucleotide regenerates the natural nucleotides. This procedure has the advantage not leaving a mark on the base and making the current sequencing cycle independent on the previous one. A more detailed description of the CoolMPS method and procedures is available under (4). Base calling and generation of FastQ files on the DNBSEQ-G400 was performed using the software release for CoolMPS (BasecallLite version_1.0.7.84).

snRNA-seq data for the native and converted libraries are available under GEO ID GSE150284 (https://www.ncbi.nlm.nih.gov/geo/query/acc.cgi?acc=GSE150284).

### snRNA-seq mapping, nuclei calling and quality filtering

Throughout the whole manuscript we refer to the index read—containing cell barcodes—as ‘read 1’, while the read containing the information of the actual cDNA fragment as ‘read 2’.

Raw sequence reads for each sample and dataset were downsampled to 2 × 200 million PE reads in a PE read-sensitive manner using the seqtk toolkit (v1.3, parameters: sample -s100 <fwd_reads.fq.gz> 200000000) before mapping reads using the mapping software zUMIs (24) (v2.5, relevant parameters: sequence_files: file1: base_definition: -BC (1–16) -UMI (17–27) file2: base_definition: -cDNA(1–91); filter_cutoffs: numb_bases:1 phred: 20 UMI_filter: num_baes: 1 phred: 20; barcodes: barcode_num: 40000 barcode_file: null automatic: yes BarcodeBinning: 1 nReadsperCell: 100 counting_opts: introns:yes downsampling: 0 strand: 0 Ham_Dist: 0 write_ham: no primaryHit: yes twoPass: yes read_layout: SE). We supplied the current version of the mm10 reference genome and gencode annotation (vM22) for the mapping. We targeted for each sample and dataset the top 40 000 cell barcodes, and counted reads in introns and exons, given the considerable amount of intronic reads in nuclear transcripts (following the official 10X guidelines).

https://support.10xgenomics.com/single-cell-gene-expression/software/pipelines/latest/advanced/references). Read duplication levels were computed with FASTQC 0.11.8. The error rate per base was estimated with the average mismatch per base reported by STAR (2.7.3a).

Data visualization and analysis were performed using SeqMonk (http://www.bioinformatics.babraham.ac.uk/projects/seqmonk/), custom RStudio (https://www.rstudio.com/) scripts and the following software packages: SoupX (v1.2.1) (25), muscat (26), Deseq2 (27), Seurat (v3.1) (28), Singlecellexperiment (29), DropletUtils (30) and org.Mm.eg.db.

For analyzing the distribution of reads in 10X libraries, we pooled mapped reads within each sample to generate four ‘pseudobulk’ .bam files. Genes were binned into 100 bins each, with the bin-size being adjusted so each bin has the same size for a given gene. We counted the number of reads mapping to each bin and calculated for each gene the relative enrichment of reads falling into the last bin versus a random bin from within the same gene. For each sample, we repeated this analysis 1000 times to estimate the average count enrichment over the last bins.

Cells/nuclei were called using the defaultDrops function (parameters: expected = 7000, upper.quant: 0.98, lower.prop = 0.1), and the non-called droplets were provided to SoupX for estimation of contaminating ambient RNA (parameters: soupRange = c(0, 1100)). For visualisation of ambient RNA contamination (Supplementary Figure S6c, d) we conducted a cell embedding using Seurat's standard workflow. Data normalization, scaling and identification of variable genes was performed using Seurat's built-in vst method with 2000 features to select. A shared-nearest-neighbours graph was constructed based on the first 10 Principal component (PC) dimensions before clustering cells using Seurat's built-in FindClusters function with a resolution of 0.4 and default parameters. SoupX requires a set of reference genes specific to cell types to estimate the present contamination (25). Using our knowledge of well-established cell marker genes and SoupX’s visualization tools to help with marker selection, we selected a short list of gene sets and provided these to SoupX’s estimateNonExpressingCells function (Mature Oligodendrocytes = *Plp1*, *Mog, Pde4b*, *St18*, *Slc24a2*, *Pcdh9*; choroid plexus cells = *Ttr*, *Htr2c*; Astrocytes = *Apoe*, *Slc1a2, Slc1a3*, *Prex2*, *Cst3*, *Gabrb1*, *Gpc5*; Neurons = *Lsamp*, *Kcnip4, Tenm2*, *Nrg3*, *Celf2*, *Grin2a*). SoupX was used to estimate and remove ambient RNA contamination for every sample individually and the results were verified using SoupX’s visualisation tools.

Finally, after inspecting sample-wise distributions of number of genes, number of UMIs and mitochondrial read proportions, we excluded cells expressing fewer than 600 or >3500 genes, as well as cells exhibiting mitochondrial read proportions higher than 0.5%.

### Cell embedding, clustering and cell type identification

For the native and converted dataset separately, we integrated all four sample-wise datasets (two from young and two from old mice), using Seurat's built-in SCTransform and integration workflow (28) (vars.to.regress = c(‘nCount_RNA’, ‘nFeature_RNA’), with 3000 genes set as integration features. Integrated datasets were then used as input for cell embedding and clustering. A shared-nearest-neighbours graph was constructed using the first 12 PC dimensions before clustering cells using Seurat's built-in FindClusters function with a resolution of 0.4 and default parameters. Umaps and tSNEs were calculated using Seurat's built-in functions, based on the first 12 PC dimensions. Count data was subsequently normalised and scaled to allow for visualisation of expression values and differential gene expression analysis. Seurat's FindAllMarkers function was run with default parameters to identify cluster markers.

For the integrated dataset (Figure 4), we repeated the analysis with the aforementioned workflow and parameters, using all eight sample-wise datasets (from each library type, two young mice and 2-old mice).

### Annotating anatomical origin of neuronal clusters

Marker genes for neuronal clusters were ranked by fold change and we obtained normalized expression data from publicly available bulk RNA-seq datasets for the top 30 markers for each cluster (23). The reference dataset was generated from pyramidal and granule cells of distinct anatomical regions of the hippocampus (dorsal/ventral DG, dorsal/ventral CA1, dorsal CA2, dorsal/ventral CA3), and contains three biological replicates for each population and region. Gene-wise scaled expression data was used as input for the hierarchical clustering. We annotated clusters depending on the enrichment of marker genes for a given brain region. This way, we were able to annotate clusters 0, 3, 5, 8 and 11.

### Pseudobulk and replicate-sensitive differential gene expression analysis

Current differential gene expression methods implemented in Seurat cannot account for biological replicates. Bulk RNA-seq methods, such as Deseq2, take replicate information into account and can perform with three or even two replicates (26,27,31). Therefore, we summarized the expression matrices for each cell type separately using muscat's aggregateData function with default parameters (26). Due to the relatively low sample power and overall shallow coverage of pseudobulk data, we decided to call differentially expressed genes at adjusted *P*-values <0.1, which is consistent with the muscat manuscript and original Deseq2 publication (26,27).

## RESULTS

### Generating CoolMPS compatible snRNA-seq libraries

**Figure 1.**
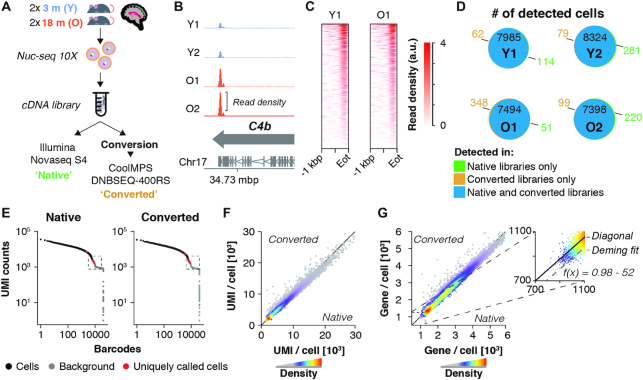
Performance of native and CoolMPS-compatible snRNA-seq libraries. (**A**) Experiment outline. Four 10 × 3′ snRNA-seq libraries were generated, derived from hippocampi of two young (3 months) and two old mice (18 months). Libraries were sequenced directly using an Illumina Novaseq or chemically converted and sequenced using CoolMPS technology on a DNBSEQ-400RS. Each library was down-sampled to contain 200 mio. paired-end reads per sample and library type. (**B**) Distribution of aggregated reads from the converted libraries dataset over C4b gene locus, separated by sample. Arrows indicate gene orientation; merged mRNA structure is depicted below. (**C**) Aligned read distribution of aggregated reads from the converted libraries dataset over the last kilobase pair of all expressed genes (*n* = 11 132 genes with rpkm > 1 in two or more samples). (**D**) Venn diagrams depict the number of cells detected in each dataset, split by samples (Y1, Y2, O1, O2). Cells of the same sample exhibiting identical cell barcodes in both datasets are considered identical cells detected by both Illumina and CoolMPS sequencing. Number of cells detected only in the native or converted libraries are colored in green and ochre, respectively. (**E**) Knee plot of sample Y1, showing UMI counts per cell barcode. Black points represent cells, with cells called only in one library type colored in red. The regions at the cutoff between called cells and empty droplets (labelled ‘background’; marked by the dashed box) are visualized at larger scale in Supplementary Figure S1b. (F, G) Scatterplot showing the cell-wise number of (**F**) detected UMIs and (**G**) genes by either library type. Insert shows the zoomed region with Deming regression line, showing a mild offset from the diagonal. Abbreviations: Y1, Y2, young male replicates 1 and 2; O1, O2, old male replicates 1 and 2; Eot, end of transcript; a.u., arbitrary unit; mbp/kbp/bp, megabase pair/kilobase pair/base pair.

We aimed to assess the quality and usability of Illumina-compatible, single-cell gene expression libraries that were chemically converted to be compatible with CoolMPS technology. To this end, we generated snRNA-seq libraries using standard Chromium 10X Single Cell 3′ reagents (version 3 chemistry) and protocols. To compare the platforms in a challenging experimental setting, we generated snRNA-seq data from frozen whole hippocampus tissues of two young and 2-old mice (3 and 18 months of age, respectively; targeting 7500 nuclei/sample) (Figure 1A). To increase the level of difficulty, we used a nuclei isolation protocol with minimal cleanup steps, which ensured an encompassing sampling of the broad population of hippocampal cells while accepting higher levels of debris and ambient RNA as a tradeoff. Thus, we generated a high-complexity dataset that we then used to assess the performance of CoolMPS across criteria like sample-wise signal-to-noise ratios, the ability to resolve finer distinction of cells and the detection of inter- and intra-replicate differences.

Aliquots of the libraries were sequenced as-is on an Illumina S4 Novaseq system, within a single lane (herein referred to as ‘native libraries’). Different aliquots of the same library stock were pooled and converted to be CoolMPS-compatible (see Materials and Methods for details) before sequenced across four lanes of a single flow cell on a DNBSEQ-G400 system (herein referred to as ‘converted’ or ‘CoolMPS-compatible’ libraries). Data from both sequencing runs were downsampled to 200 million reads per sample, trimmed and mapped with identical settings.

### CoolMPS-compatible libraries maintain distinct hallmarks of droplet-based sequencing assays

Droplet-based RNA-seq methods such as 10X rely on capturing mRNA molecules through their polyA-tail, thus enriching predominantly fragments derived from the 3′ end (6). Indeed, we observed an enrichment of reads at the 3′ end of genes (exemplified by C4b, Figure 1B). We binned each gene into 100 bins and observed that the last bin prior the 3′ end exhibited an average 5-fold enrichment over any other bin of the same gene (native libraries: mean 5.4-fold ± 0.8 standard deviation; converted libraries: mean 5.2-fold ± 1 standard deviation). Fifteen percent of all reads mapping to genes (native libraries: mean 15.6% ± 0.8% standard deviation; converted libraries: mean 14.6% ± 0.3% standard deviation) fell within a 300–350 bp region located at the annotated end of transcripts, where we observed the highest read densities (Eot, Figure 1C; Supplementary Figure S1a). Consistently, data from both native and converted libraries exhibited a 98% overlap of detected cells (Figure 1D), merely differing by a few hundred cells at the end of the count-barcode distribution (Figure 1E; Supplementary Figure S1b). Furthermore, commonly detected cells were correlated with respect to the number of detected UMIs and genes (*R*^2^ = 0.98 and *R*^2^ = 0.97, respectively) (Figure 1F, G), although Deming regression indicated a small offset of ∼50–80 genes per cell for converted libraries. This might be related to a slightly lowered fraction of reads with a mean Q30 value, which we observed to drop from 98% to 95% and 96% to 91% on reads 1 and 2, respectively (Supplementary Figure S2a–k). However, we found no evidence that any particular group of transcripts, genes or pathways was affected. Furthermore, we performed analysis of conventional metrics including mapping rate (Supplementary Figure S2j), error rate (Supplementary Figure S2k), duplication levels (Supplementary Figure S3a–h), GC content (Supplementary Figure S4a–i) and base composition per cycle (Supplementary Figure S5a, b). We noted that the estimated error rate was twice as high among the converted libraries, increasing from an average of 0.19% (native libraries) to 0.41% (converted libraries). This level of error rate is not expected to notably affect the overall results for single-cell transcriptomics. The remaining quality metrics provided no further evidence that DNB-conversion and sequencing with CoolMPS had other negative impacts on overall library quality. Thus, the conversion reaction preserved the overall structure and composition of a droplet-based snRNA-seq dataset.

### Detection and removal of known contaminants is unaffected by conversion reaction

scRNA-seq data is known to be affected by contaminants such as damaged cells (characterized by high mitochondrial read proportions) or ambient, cell-free RNA, which can compromise the transcriptome of intact cells and falsely indicate expression of non-endogenous genes in a given cell population (5,25). Considering this, we asked if the conversion reaction would negatively impact our analysis’ ability to distinguish nuanced biological signals from noise. Given that our libraries were generated from single nuclei, only 8% of the nuclei detected from the native libraries and 7% from the converted libraries, exhibited mitochondrial read proportions higher than 0.5%, and were excluded from further analyses (Supplementary Figure S6a). As expected, there was a highly significant (*P*-value < 10^−3^), 66% overlap of excluded nuclei among the native and CoolMPS-compatible libraries (Supplementary Figure S6b). Among many of the cells passing this first filtering step, we detected low levels of transcripts usually unique to choroid plexus, such as transthyretin (Ttr; Supplementary Figure S6c, d). This phenomenon is due to RNA derived from damaged cells, that is included in droplets of intact nuclei. We detected that this contamination—sometimes referred to as 'soup' (25)—varied among samples but was not statistically different across library types (Supplementary Figure S6e). After correcting the expression matrices for ambient RNA contamination, we still found high correlation between native and converted libraries with respect to the number of UMIs and genes per nuclei (*R*^2^ = 0.979 and *R*^2^ = 0.972, respectively) (Supplementary Figure S6f, g). Taken together, the library conversion did not affect accurate differentiation between signal and noise.

### Equivalent identification of cell populations across library types

**Figure 2.**
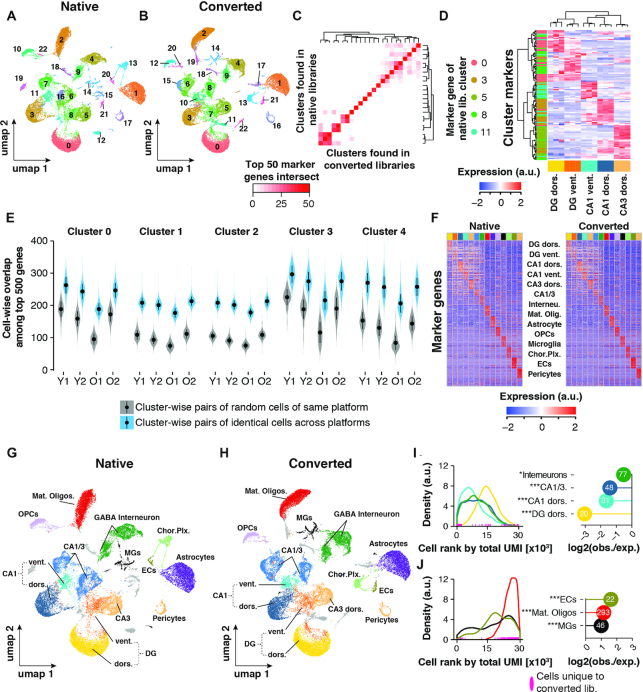
Cell clustering performance across libraries. (A, B) UMAP representation of cells and clustering results of (**A**) *n* = 29 945 cells (native libraries) and (**B**) *n* = 30 360 (converted libraries), respectively. (**C**) Heatmap depicting the number of common top 50 genes per cluster as detected in either sequencing run. (**D**) Identification of spatial origin of five neuronal clusters. We performed hierarchical clustering of the top 30 marker genes (rows) of clusters 0, 3, 5, 8 and 11 as identified in the native dataset. For those marker genes, expression is plotted as measured in a publicly available RNA-seq data of anatomically distinct neurons (columns; *n* = 3 replicates per neuronal population). Cells within the heatmap are colored to represent row-wise z-transformed expression of given gene across all anatomically distinct neurons. Marker genes are annotated by the row-side color bar, with each marker gene colored by cluster. Expression data was derived from (Cembrowski *et al.* 2016). (**E**) Cluster and sample-wise distribution of common top 500 expressed genes between two cells. Cluster number corresponds to the clusters found in the native dataset in (A). Gray violins represent overlaps from cells of the same cluster (biological cell replicates); blue violins represent overlaps from cells with the same barcode, sequenced in both datasets (technical replicates). Means ± SEM. (**F**) Relative expression of top 30 cell marker genes as found in native library dataset. Each column represents *n* = 50 of randomly selected cells per cell type. (G, H) UMAP representation of cells with annotated cell types in (**G**) native and (**H**) converted library dataset, respectively. (I, J) Analysis of depletion (**I**) and enrichment (**J**) of cells found only in the CoolMPS-compatible dataset among neuronal and non-neuronal cell populations. Left-hand panels: Distribution of cell types among all cells, ranked by their total UMI count in descending order (converted libraries only; cell with the highest total UMI count has rank 0). Rank of cells for given cell types that were found only in the CoolMPS-compatible dataset are indicated along the x-axes. Right-hand panels: Y-axis indicates for each cell type the observed-over-expected values for cells that were found only in the CoolMPS-compatible dataset. Absolute number of cells found only in the CoolMPS-compatible dataset are indicated in the bubble. Cell types exhibiting no statistical enrichment or depletion for CoolMPS-unique cells are not shown. ***P_adj_ < 0.001, **P_adj_ < 0.01, *P_adj_ < 0.05, two-sided Fisher's exact test, adjusted for multiple testing. Abbreviations: Y1, Y2, Young male replicates 1 and 2; O1, O2, Old male replicates 1 and 2; DG dors., granule neurons of dorsal dentate gyrus; DG vent., granule neurons of ventral dentate gyrus; CA1 dors., pyramidal neurons of dorsal CA1; CA1 vent., pyramidal neurons of ventral CA1; CA3 dors., pyramidal neurons of dorsal CA3; CA1/CA3 Neur., pyramidal neurons that could not be clearly allocated to CA1 and CA3; GABA interneuron, Gabaergic interneurons; Mat. Oligos, mature oligodendrocytes; OPCs, oligodendrocyte precursors; MGs, microglia; Chor. Plx., choroid plexus cells; ECs, brain endothelial cells; a.u., arbitrary unit; obs./exp., observed over expected.

We next asked if clustering and detection of cell types would be affected by the conversion reaction and/or subsequent sequencing with a different instrument. Using identical parameters for the analysis, both datasets yielded consistent results; 23 clusters were identified for each dataset and the clusters could be unambiguously associated across datasets by simply intersecting their respective top 50 marker genes (Figure 2A–C). Interestingly, we discovered in both datasets multiple clusters of neuronal cells that were located adjacent to one another when visualized via umaps. Following recent publications deriving spatial origin of neurons from bulk and single-cell transcriptomes (23,32), we performed hierarchical clustering of the top 30 marker genes for each population using publicly available RNA-seq data of anatomically distinct neurons in the hippocampus (23). The analysis revealed five clusters that could be clearly identified: granule cells of the ventral and dorsal DG, and pyramidal neurons of dorsal and ventral CA1 and dorsal CA3 (Figure 2D). There were also three clusters of pyramidal cells, which we could not clearly anatomically identify and were thus labelled as CA1/3.

While we could not identify any significant impact of the library conversion or sequencing method on cell clustering, i.e. on the scale of cell populations, we next asked whether the transcriptomes of individual cells might have been compromised. To this end, we calculated for every nucleus, in each dataset separately, the 500 highest expressed genes. We then identified the overlap of these 500 genes for nuclei present in both datasets (‘pair of identical cells across platforms’), thus representing technical reproducibility of the single-nuclei transcriptome across library types. As a comparison, we chose the biological reproducibility, i.e. the similarity of single-nuclei transcriptomes from two random cells of the same cell type/cluster within the native library dataset (‘pair of random cells of same platform’). Across every sample and cluster, the technical reproducibility outperformed the biological reproducibility (represented by the five largest clusters; Figure 2E). We thus found no evidence that the conversion reaction significantly altered the single-nucleus transcriptome.

Finally, we annotated all detected clusters, and found all major cell types and cell populations previously detected in the hippocampus among both datasets (Figure 2F–H) (15). (Figure 2F–H). Interestingly, cells detected only in the CoolMPS-compatible libraries were significantly under-represented in neuronal cell populations, but enriched in oligodendrocytes, microglia and endothelial cells (Figure 2I, J). Non-neuronal cells tend to express overall fewer transcripts, which can be visualized by ranking individual cells by their total number of UMIs and plotting the distribution of cell types along this ranking (Figure 2I, J). Given that cells detected only in the CoolMPS-compatible libraries were enriched toward the end of this count-barcode distribution (compare Figure 1E), their overrepresentation among non-neuronal cells is expected.

In summary, the selected nuclei isolation protocol allowed sampling and identification of multiple neuronal and non-neuronal cell populations at fine resolution. Moreover, library conversion followed by sequencing using antibody-based nucleotide labeling did not affect the ability to resolve even fine distinction of cells.

### Conversion reaction preserves inter-replicate variance

**Figure 3.**
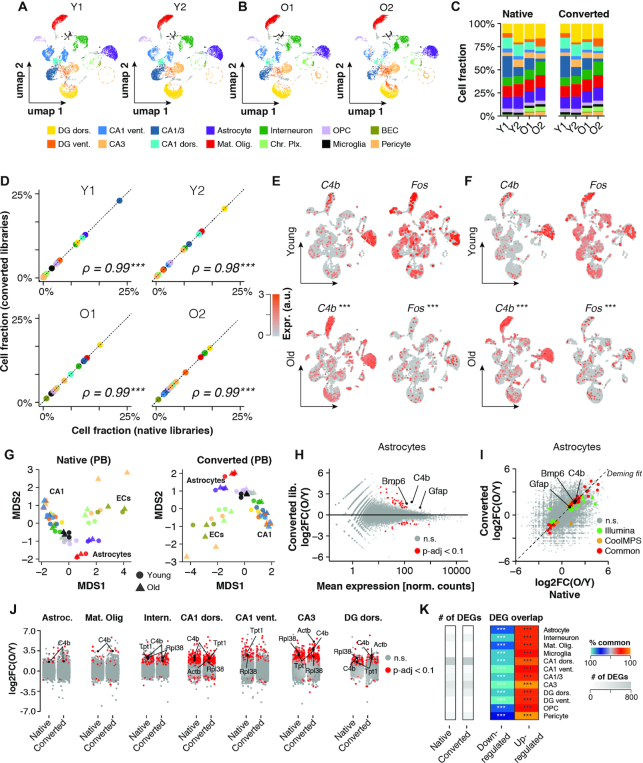
Inter- and intra-replicate differences are unaffected by conversion reaction. (A, B) UMAP representation of cells (CoolMPS-compatible dataset) with annotated cell types split by replicates across (**A**) young and (**B**) old. Circles highlight differential abundance of choroid plexus-derived cells and Pericytes. (**C**) Relative cell composition across replicates and datasets. (**D**) Sample-wise correlation of cell fractions as detected in the native and converted libraries dataset. ***P < 0.001, **P < 0.01, *P < 0.05, Spearman rank correlation test. (E, F) Expression levels of C4b and Fos per cell colored on UMAP as detected in (**E**) the native dataset and (**F**) the CoolMPS-compatible dataset, respectively. Expression of both genes changes significantly with age across all cells. ***P_adj_ < 0.001, **P_adj_ < 0.01, *P_adj_ < 0.05, Two-sided Wilcoxon rank-sum test, adjusted for multiple testing. (**G**) Pseudobulk-level Multidimensional Scaling (MDS) plot for both datasets. Each point represents one cell population in a given sample. Points are colored by cell population and shaped by age. (**H**) Pseudobulk-level MA-plot for Astrocyte population (CoolMPS-compatible dataset). Genes showing differential expression (*P*_adj_ < 0.1) between old and young are marked in red. *P_adj_ < 0.1, two-sided Wald test, adjusted for multiple testing. (**I**) Cross-platform correlation of pseudobulk-level, gene-wise log_2_-transformed expression ratios between young and old samples. Detected DEGs (*P*_adj_ < 0.1) common and distinct to either dataset are colored. (**J**) Distribution of pseudobulk-level gene-wise expression changes between old and young animals, resolved by cell population. Genes showing differential expression (*P*_adj_ < 0.1) between old and young in given cell type are marked in red. (**K**) Total number of detected DEGs per cell population using either the native or converted libraries (grey heatmap) and per-cell population overlap of common DEGs relative to total DEGs, split by up- and down-regulated genes. % overlap is indicated by color scale. ***P_adj_ < 0.001, **P_adj_ < 0.01, *P_adj_ < 0.05, two-sided Fisher's exact test, adjusted for multiple testing. Abbreviations: Y1, Y2, young male replicates 1 and 2; O1, O2, old male replicates 1 and 2; DG dors., granule neurons of dorsal dentate gyrus; DG vent., granule neurons of ventral dentate gyrus; CA1 dors., pyramidal neurons of dorsal CA1; CA1 vent., pyramidal neurons of ventral CA1; CA3 dors., pyramidal neurons of dorsal CA3; CA1/CA3 Neur., pyramidal neurons that could not be clearly allocated to CA1 and CA3; GABA interneuron, GABAergic interneurons; Mat. Oligos, mature oligodendrocytes; OPCs, oligodendrocyte precursors; MGs, microglia; Chor. Plx., choroid plexus cells; ECs, brain endothelial cells; Astroc., astrocytes; n.s., not statistically significant; a.u., arbitrary unit; obs./exp., observed over expected.

In order to determine the robustness of the conversion protocol across multiple samples, we next analyzed if differences within and between sample replicates of the same age group would be unaffected in CoolMPS-compatible libraries. First, we inspected possible differences in cellular composition with age. We detected an increased proportion of pericytes and cells of the choroid plexus in older animals (Figure 3A–C). Given that the choroid plexus is an anatomically distinct region of the brain and not part of the hippocampus, we assumed that the higher abundance of these cells result from increased fibrosis (33), ultimately lowering tissue dissociation quality (34). Data from CoolMPS-compatible libraries corroborated higher levels of choroid plexus cells. Moreover, we observed that relative cell abundance levels across cell types and replicates were close to identical in both datasets (Figure 3D), confirming that the conversion reaction yielded robust performance across samples.

One of the key analyses in RNA-seq benefiting from biological replicates is the detection differential gene expression (DGE) (31). Consistent with multiple studies that performed bulk and single-cell DGE analyses of the aging brain, we observed in both datasets increased expression of inflammatory markers such as *C4b*, and decreased levels of *Fos*, an indicator of neuronal activity (Figure 3e) (19,35–37). Current software packages for DGE analyses of single cells are not replicate-sensitive, but this limitation can be overcome by pseudobulk (PB) analyses (26). Across cell types, PB DGE analyses using the *DESeq2* software package (27) resulted in highly consistent results in both the native and CoolMPS-compatible dataset (Figure 3G–K). As such, we observed a strong correlation of expression fold changes (exemplified by Astrocytes; Figure 3I), and a highly significant overlap of common differentially expressed genes (Figure 3I–K). On average, 65% of the significantly down-regulated genes and 60% of significantly up-regulated genes were detected in both datasets. We observed the highest overlap among neurons of the CA3 region (79%) and the lowest among neurons of the ventral CA1 region (52%). Consistent with a previous single-cell RNA-seq analyses of aged brain tissues of mice (19), we found genes with significant up-regulation across most cell types, such as *C4b, Tpt1*, and *Actb*, and up-regulation in several genes coding for ribosomal proteins, including *Rpl38*.

Taken together, we found that chemical conversion of Illumina-compatible libraries followed by sequencing on a DNBSEQ-G400 system yielded robust results across multiple samples and preserved replicate-sensitive differences of biologically relevance.

### Data of native and CoolMPS-compatible libraries can be integrated

**Figure 4.**
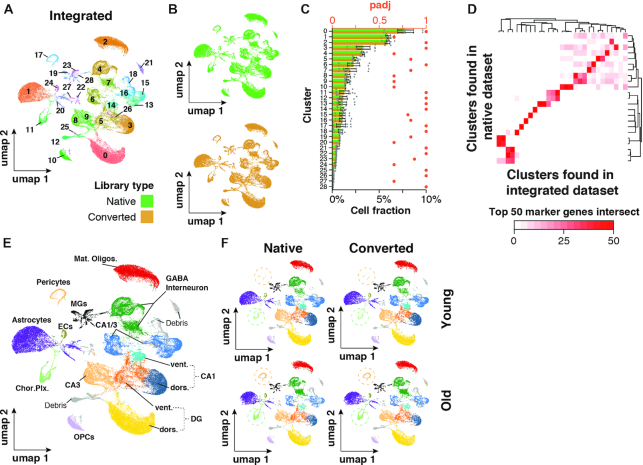
Integrated native and CooMPS-compatible snRNA-seq datasets show no separation by library type. (A, B) UMAP representation of cells and clustering results of *n* = 60,0720 cells integrated from both datasets. Cells are colored by (**A**) cluster or (**B**) library type. Cells were split by library type to improve visibility. (**C**) Relative abundance of each cluster normalized to total cells. Relative abundances are split by library type to gauge conversion-dependent composition of clusters. *n* = 4 per dataset. Means ± SEM. ***P_adj_ < 0.001, **P_adj_ < 0.01, *P_adj_ < 0.05, two-sided Wilcoxon rank-sum test, adjusted for multiple testing. Adjusted *P*-values for paired, two-sided Wilcoxon test probing differential abundance per cluster are shown in red. (**D**) Heatmap depicting the number of common top 50 genes per cluster as detected in either integrated or native dataset. (**E**, **F**) UMAP representation of cell populations from integration dataset when (A) collapsed or (B) split by dataset and age. Circles highlight differential abundance of choroid plexus-derived cells and Pericytes. Abbreviations: DG dors., granule neurons of dorsal dentate gyrus; DG vent., granule neurons of ventral dentate gyrus; CA1 dors., pyramidal neurons of dorsal CA1; CA1 vent., pyramidal neurons of ventral CA1; CA3 dors., pyramidal neurons of dorsal CA3; CA1/CA3 Neur., pyramidal neurons that could not be clearly allocated to CA1 and CA3; GABA interneuron, gabaergic interneurons; Mat. oligos, mature oligodendrocytes; OPCs, oligodendrocyte precursors; MGs, microglia; Chor. Plx., choroid plexus cells; ECs, brain endothelial cells; a.u., arbitrary unit; obs./exp., observed over expected; *P*_adj_, adjusted *P*-value.

Finally, we bioinformatically pooled both snRNA-seq datasets to test if nuclei from both library types would form coherent clusters (herein referred to as ‘integrated dataset’). To this end, we used standard dataset integration algorithms and workflows (28) and performed embedding and clustering of the nuclei similar to the analyses conducted on each dataset individually (compare Figure 2A–D). Visualized in 2D space using umaps, we observed that nuclei from both datasets clustered without any detectable technical bias (Figure 4A, B). Each of the resulting 29 clusters was equally composed of nuclei from both datasets, and the per-replicate abundance was unaffected (tested using cluster-wise paired Wilcoxon rank sum tests; Figure 4C). Most nuclei clusters found in the integrated dataset could be directly associated to clusters found in the native libraries dataset via their top 50 marker genes, with small clusters being the exception (Figure 4D). After completing nuclei annotation, we found that the integrated dataset yielded a cell composition profile with remarkable similarity to both the native and CoolMPS-compatible dataset (Figure 4E). Split by replicates, we also found that the integrated dataset maintained relevant features previously identified in each dataset separately, including increased levels of pericytes and choroid plexus-derived cells in the aged samples (Figure 4F). Thus, native and CoolMPS-compatible libraries yield highly consistent sequencing results that can be analyzed both interchangeably and combined.

## DISCUSSION

The process of sequencing has experienced little innovation in recent years compared to the explosion of its versatile applications, with the exception of long read sequencing (1,38). While overcoming several of its early limitations, long read sequencers do not yield the same throughput of current short-read sequencers (2). We have therefore taken an interest in the release of CoolMPS and corresponding conversion kits, as this technology could provide a possible addition to the existing set of sequencing platforms. The application of fluorescently labeled antibodies is a considerable novelty that could streamline the efficiency of the sequencing process since antibodies could potentially be re-used. Furthermore, sequencing via nucleotide-sensitive antibodies could eventually yield reads beyond the current 150–300 bp limit, since dye-free nucleotides are less likely to interfere with subsequent sequencing cycles (i.e. reduced ‘scarring’). However, we are not aware of peer-reviewed studies assessing the performance of chemically converted droplet-based libraries sequenced with CoolMPS.

For this study, we chose to assess the performance of converted libraries generated with the Chromium Single-cell ‘3 reagent kit (version 3), as it is currently among the most widely used droplet-based technologies, and several single-cell and single-nucleus atlas datasets have been generated with it (7,9,11,17,39,40). Conventional MGISEQ chemistry has been successfully applied to Smart-seq2 and Chromium 10X (version 2 chemistry) single-cell libraries (12,13), yet sequencing via antibody-labelled bases is fundamentally different to the classical sequencing-by-synthesis approach. Additionally, the currently distributed Chromium 10X version 3 chemistry yields significantly more complex transcriptome, doubling the number of detectable genes and transcripts per cell (14). Whether the biological and technical complexity of 10X v3 libraries is maintained during the DNB conversion process and properly resolved by sequencing with CoolMPS, is thus not evident until experimentally tested and verified. However, we acknowledge that multiple other single-cell RNA-seq technologies exist (14) and we have not assessed whether chemical conversion and sequencing with CoolMPS is applicable to these. In addition, we only tested the performance of the conversion kit for droplet-based RNA-seq libraries but not for other single-cell assays such as single-cell Atac-seq (5). Given the resources required for generating and sequencing single-cell libraries from multiple technologies and assays, we consider this to be beyond the scope of this study but expect more studies to explore the performance of CoolMPS-compatible libraries for other sequencing assays.

To test the performance of converted libraries, we generate snRNA-seq libraries from hippocampus tissue, given its diverse composition of cell types and anatomical complexity. We elected to use a previously published nuclei isolation protocol (15) that involves little physical or chemical filtering (e.g. no removal of myelin or application of density gradients) to obtain a diverse sample of present cell populations while accepting significant amounts of debris and ambient RNA. Indeed, the dataset contained all cell types associated with hippocampal adult mouse tissue (15) and contained neurons with transcriptional signatures of distinct anatomical origin (23). CoolMPS-compatible libraries recapitulate these biological features of our dataset in every aspect and detail, including cell type diversity, fine differences between cells from distinct hippocampal regions and expression changes with age. Similarly, we found no evidence that the library conversion caused a blurring between biological signal and background contaminants. Since this study focuses on the comparison of native and converted libraries, we did not examine the biological validity of cell composition or expression shifts with age. However, findings from previous single-cell studies in aged mice do align with some of the most pronounced changes found in our analyses, such as increased expression of complement protein *C4b* across cell types (19) and astrocytes adopting an activated state marked by higher expression of *Gfap* (18). We anticipate that future studies will demonstrate that the consistency between native and CoolMPS-compatible scRNA-seq libraries is not dependent on the tissue or studied perturbation.

The main difference we identified was a slightly reduced single base call quality in the CoolMPS-compatible libraries compared to native. We cannot resolve here whether this drop in per-base Q30 values is a consequence of the sequencer or the conversion. Preliminary sequencing data of libraries generated with CoolMPS-compatible reagents (i.e. no conversion was required) have suggested that the sequencing platform itself results in base call errors similar to those seen with dye-labeled nucleotides (4). In addition, conventional MGISEQ-sequencing after DNB-conversion of droplet-based libraries also reported a drop of Q30 barcodes, Q30 UMIs and Q30 RNAs by 4–6 percentage points, compared to sequencing of native libraries on an Illumina NovaSeq (12). This may indicate that the conversion reaction, which involves five extra PCR cycles, could indeed lower the libraries’ per-base quality. However, only a direct sequencing comparison of the identical libraries between all three technologies (Illumina, MGISEQ and CoolMPS)—or a conversion-free approach—would definitely resolve this issue. In addition, lowering the number of PCR cycles could thus represent a possible optimization of the protocol.

Interestingly, our analyses demonstrated that the lowered Q30 values were largely inconsequential, given the performances of native and converted libraries across all tested features. One explanation for this could be that the average base calling quality of the converted libraries was still >80%, comparable to the industry-reported performance of HiSeq 2500 instruments (41), which have been widely accepted and used in studies such as the 1000 Genomes Project (42). Furthermore, trimming of low-quality bases is common practice prior to mapping, and effective reads of only 25 bp length yield transcriptome data largely identical to the one generated with longer reads (31,43). Finally, RNA-seq libraries generated with the 10 × 3′ platform cover only a fraction of the exome (fragments of app. 350 bp at the 3′ end), which allows for quantification of gene expression but has limited usability beyond that (no splicing or genotyping analyses). Therefore, a mild reduction in base calling quality may thus be tolerable.

In conclusion, our study provides the first line of evidence that CoolMPS is a robust method for sequencing single-cell/single-nucleus RNA-seq libraries from droplet-based assays.

## DATA AVAILABILITY

The datasets supporting the conclusions of this article are available in the NCBI’s Gene Expression Omnibus repository [GEO: GSE150284].

## Supplementary Material

gkaa1127_Supplemental_Files

## References

[1] Heather J.M., Chain B. The sequence of sequencers: the history of sequencing DNA. Genomics. 2016; 107:1–8. 26554401 10.1016/j.ygeno.2015.11.003 PMC4727787

[2] Levy S.E., Myers R.M. Advancements in next-generation sequencing. Annu. Rev. Genomics Hum. Genet. 2016; 17:95–115. 27362342 10.1146/annurev-genom-083115-022413

[3] Wang Y., Mashock M., Tong Z., Mu X., Chen H., Zhou X., Zhang H., Zhao G., Liu B., Li X. Changing technologies of RNA sequencing and their applications in clinical oncology. Front. Oncol. 2020; 10:447. 32328458 10.3389/fonc.2020.00447 PMC7160325

[4] Drmanac S., Callow M., Chen L., Zhou P., Eckhardt L., Xu C., Gong M., Gablenz S., Rajagopal J., Yang Q. et al. CoolMPS^TM^: advanced massively parallel sequencing using antibodies specific to each natural nucleobase. 2020; bioRxiv doi:20 February 2020, preprint: not peer reviewed 10.1101/2020.02.19.953307.

[5] Amezquita R.A., Lun A.T.L., Becht E., Carey V.J., Carpp L.N., Geistlinger L., Marini F., Rue-Albrecht K., Risso D., Soneson C. et al. Orchestrating single-cell analysis with Bioconductor. Nat. Methods. 2020; 17:137–145. 31792435 10.1038/s41592-019-0654-x PMC7358058

[6] Zheng G.X.Y., Terry J.M., Belgrader P., Ryvkin P., Bent Z.W., Wilson R., Ziraldo S.B., Wheeler T.D., McDermott G.P., Zhu J. et al. Massively parallel digital transcriptional profiling of single cells. Nat. Commun. 2017; 8:14049. 28091601 10.1038/ncomms14049 PMC5241818

[7] Tabula Muris Consortium, Overall coordination, Logistical coordination, Organ collection and processing, Library preparation and sequencing, Computational data analysis, Cell type annotation, Writing group, Supplemental text writing group and Principal investigators Single-cell transcriptomics of 20 mouse organs creates a Tabula Muris. Nature. 2018; 562:367–372. 30283141 10.1038/s41586-018-0590-4 PMC6642641

[8] Han X., Zhou Z., Fei L., Sun H., Wang R., Chen Y., Chen H., Wang J., Tang H., Ge W. et al. Construction of a human cell landscape at single-cell level. Nature. 2020; 581:309–303. 10.1038/s41586-020-2157-4 32214235

[9] Mathys H., Davila-Velderrain J., Peng Z., Gao F., Mohammadi S., Young J.Z., Menon M., He L., Abdurrob F., Jiang X. et al. Single-cell transcriptomic analysis of Alzheimer's disease. Nature. 2019; 570:332–337. 31042697 10.1038/s41586-019-1195-2 PMC6865822

[10] Ma S., Sun S., Geng L., Song M., Wang W., Ye Y., Ji Q., Zou Z., Wang S., He X. et al. Caloric Restriction Reprograms the Single-Cell Transcriptional Landscape of Rattus Norvegicus Aging. Cell. 2020; 180:984–1001. 32109414 10.1016/j.cell.2020.02.008

[11] Schaum N., Lehallier B., Hahn O., Pálovics R., Hosseinzadeh S., Lee S.E., Sit R., Lee D.P., Losada P.M., Zardeneta M.E. et al. Ageing hallmarks exhibit organ-specific temporal signatures. Nature. 2020; 583:596–602. 32669715 10.1038/s41586-020-2499-y PMC7757734

[12] Natarajan K.N., Miao Z., Jiang M., Huang X., Zhou H., Xie J., Wang C., Qin S., Zhao Z., Wu L. et al. Comparative analysis of sequencing technologies for single-cell transcriptomics. Genome Biol. 2019; 20:70. 30961669 10.1186/s13059-019-1676-5 PMC6454680

[13] Senabouth A., Andersen S., Shi Q., Shi L., Jiang F., Zhang W., Wing K., Daniszewski M., Lukowski S.W., Hung S.S.C. et al. Comparative performance of the BGI and Illumina sequencing technology for single-cell RNA-sequencing. NAR Genom Bioinform. 2020; 2:lqaa034. 10.1093/nargab/lqaa034 PMC7671348 33575589

[14] Ding J., Adiconis X., Simmons S.K., Kowalczyk M.S., Hession C.C., Marjanovic N.D., Hughes T.K., Wadsworth M.H., Burks T., Nguyen L.T. et al. Systematic comparison of single-cell and single-nucleus RNA-sequencing methods. Nat. Biotechnol. 2020; 38:737–746. 32341560 10.1038/s41587-020-0465-8 PMC7289686

[15] Habib N., Avraham-Davidi I., Basu A., Burks T., Shekhar K., Hofree M., Choudhury S.R., Aguet F., Gelfand E., Ardlie K. et al. Massively parallel single-nucleus RNA-seq with DroNc-seq. Nat. Methods. 2017; 14:955–958. 28846088 10.1038/nmeth.4407 PMC5623139

[16] Keren-Shaul H., Spinrad A., Weiner A., Matcovitch-Natan O., Dvir-Szternfeld R., Ulland T.K., David E., Baruch K., Lara-Astaiso D., Toth B. et al. A unique microglia type associated with restricting development of Alzheimer's disease. Cell. 2017; 169:1276–1290. 28602351 10.1016/j.cell.2017.05.018

[17] Zhou Y., Song W.M., Andhey P.S., Swain A., Levy T., Miller K.R., Poliani P.L., Cominelli M., Grover S., Gilfillan S. et al. Human and mouse single-nucleus transcriptomics reveal TREM2-dependent and TREM2-independent cellular responses in Alzheimer's disease. Nat. Med. 2020; 26:131–142. 31932797 10.1038/s41591-019-0695-9 PMC6980793

[18] Habib N., McCabe C., Medina S., Varshavsky M., Kitsberg D., Dvir-Szternfeld R., Green G., Dionne D., Nguyen L., Marshall J.L. et al. Disease-associated astrocytes in Alzheimer's disease and aging. Nat. Neurosci. 2020; 23:701–706. 32341542 10.1038/s41593-020-0624-8 PMC9262034

[19] Ximerakis M., Lipnick S.L., Innes B.T., Simmons S.K., Adiconis X., Dionne D., Mayweather B.A., Nguyen L., Niziolek Z., Ozek C. et al. Single-cell transcriptomic profiling of the aging mouse brain. Nat. Neurosci. 2019; 22:1696–1708. 31551601 10.1038/s41593-019-0491-3

[20] Dulken B.W., Buckley M.T., Navarro Negredo P., Saligrama N., Cayrol R., Leeman D.S., George B.M., Boutet S.C., Hebestreit K., Pluvinage J.V. et al. Single-cell analysis reveals T cell infiltration in old neurogenic niches. Nature. 2019; 571:205–210. 31270459 10.1038/s41586-019-1362-5 PMC7111535

[21] Gate D., Saligrama N., Leventhal O., Yang A.C., Unger M.S., Middeldorp J., Chen K., Lehallier B., Channappa D., De Los Santos M.B. et al. Clonally expanded CD8 T cells patrol the cerebrospinal fluid in Alzheimer's disease. Nature. 2020; 577:399–404. 31915375 10.1038/s41586-019-1895-7 PMC7445078

[22] Pluvinage J.V., Wyss-Coray T. Systemic factors as mediators of brain homeostasis, ageing and neurodegeneration. Nat. Rev. Neurosci. 2020; 21:93–102. 31913356 10.1038/s41583-019-0255-9

[23] Cembrowski M.S., Wang L., Sugino K., Shields B.C., Spruston N. Hipposeq: a comprehensive RNA-seq database of gene expression in hippocampal principal neurons. Elife. 2016; 5:e14997. 27113915 10.7554/eLife.14997 PMC4846374

[24] Parekh S., Ziegenhain C., Vieth B., Enard W., Hellmann I. zUMIs - a fast and flexible pipeline to process RNA sequencing data with UMIs. Gigascience. 2018; 7:giy059. 10.1093/gigascience/giy059 PMC6007394 29846586

[25] Young M.D., Behjati S. SoupX removes ambient RNA contamination from droplet based single-cell RNA sequencing data. 2020; bioRxiv doi:03 February 2020, preprint: not peer reviewed 10.1101/303727. PMC7763177 33367645

[26] Crowell H.L., Soneson C., Germain P.-L., Calini D., Collin L., Raposo C., Malhotra D., Robinson M.D. On the discovery of population-specific state transitions from multi-sample multi-condition single-cell RNA sequencing data. 2019; bioRxiv doi:26 July 2019, preprint: not peer reviewed 10.1101/713412. PMC7705760 33257685

[27] Love M.I., Huber W., Anders S. Moderated estimation of fold change and dispersion for RNA-seq data with DESeq2. Genome Biol. 2014; 15:550. 25516281 10.1186/s13059-014-0550-8 PMC4302049

[28] Stuart T., Butler A., Hoffman P., Hafemeister C., Papalexi E., Mauck W.M. 3rd, Hao Y., Stoeckius M., Smibert P., Satija R. Comprehensive Integration of Single-Cell Data. Cell. 2019; 177:1888–1902. 31178118 10.1016/j.cell.2019.05.031 PMC6687398

[29] Lun A., Risso D., Korthauer K. SingleCellExperiment: S4 classes for single cell data. 2018; R package version 1.

[30] Lun A.T.L., Riesenfeld S., Andrews T., Dao T.P., Gomes T., participants in the 1st Human Cell Atlas Jamboree, Marioni J.C. EmptyDrops: distinguishing cells from empty droplets in droplet-based single-cell RNA sequencing data. Genome Biol. 2019; 20:63. 30902100 10.1186/s13059-019-1662-y PMC6431044

[31] Conesa A., Madrigal P., Tarazona S., Gomez-Cabrero D., Cervera A., McPherson A., Szcześniak M.W., Gaffney D.J., Elo L.L., Zhang X. et al. A survey of best practices for RNA-seq data analysis. Genome Biol. 2016; 17:13. 26813401 10.1186/s13059-016-0881-8 PMC4728800

[32] Habib N., Li Y., Heidenreich M., Swiech L., Avraham-Davidi I., Trombetta J.J., Hession C., Zhang F., Regev A. Div-Seq: single-nucleus RNA-Seq reveals dynamics of rare adult newborn neurons. Science. 2016; 353:925–928. 27471252 10.1126/science.aad7038 PMC5480621

[33] Wegiel J., Kuchna I., Wisniewski T., de Leon M.J., Reisberg B., Pirttila T., Kivimaki T., Lehtimaki T. Vascular fibrosis and calcification in the hippocampus in aging, Alzheimer disease, and Down syndrome. Acta Neuropathol. 2002; 103:333–343. 11904752 10.1007/s00401-001-0471-y

[34] Stankiewicz A.M., Goscik J., Majewska A., Swiergiel A.H., Juszczak G.R. The effect of acute and chronic social stress on the hippocampal transcriptome in mice. PLoS One. 2015; 10:e0142195. 26556046 10.1371/journal.pone.0142195 PMC4640871

[35] Kitraki E., Bozas E., Philippidis H., Stylianopoulou F. Aging-related changes in IGF-II and c-fos gene expression in the rat brain. Int. J. Dev. Neurosci. 1993; 11:1–9. 7683839 10.1016/0736-5748(93)90029-d

[36] Villeda S.A., Plambeck K.E., Middeldorp J., Castellano J.M., Mosher K.I., Luo J., Smith L.K., Bieri G., Lin K., Berdnik D. et al. Young blood reverses age-related impairments in cognitive function and synaptic plasticity in mice. Nat. Med. 2014; 20:659–663. 24793238 10.1038/nm.3569 PMC4224436

[37] Schaum N., Lehallier B., Hahn O., Hosseinzadeh S., Lee S.E., Sit R., Lee D.P., Losada P.M., Zardeneta M.E., Pálovics R. et al. Ageing hallmarks exhibit organ-specific temporal signatures. Nature. 2019; 583:596–602. 10.1038/s41586-020-2499-y PMC7757734 32669715

[38] Amarasinghe S.L., Su S., Dong X., Zappia L., Ritchie M.E., Gouil Q. Opportunities and challenges in long-read sequencing data analysis. Genome Biol. 2020; 21:30. 32033565 10.1186/s13059-020-1935-5 PMC7006217

[39] Kimmel J.C., Penland L., Rubinstein N.D., Hendrickson D.G., Kelley D.R., Rosenthal A.Z. Murine single-cell RNA-seq reveals cell-identity- and tissue-specific trajectories of aging. Genome Res. 2019; 29:2088–2103. 31754020 10.1101/gr.253880.119 PMC6886498

[40] Tabula Muris Consortium A single-cell transcriptomic atlas characterizes ageing tissues in the mouse. Nature. 2020; 583:590–595. 32669714 10.1038/s41586-020-2496-1 PMC8240505

[41] HiSeq 2500 Specifications | Key performance parameters.

[42] 1000 Genomes Project Consortium Auton A., Brooks L.D., Durbin R.M., Garrison E.P., Kang H.M., Korbel J.O., Marchini J.L, McCarthy S., McVean G.A. et al. A global reference for human genetic variation. Nature. 2015; 526:68–74. 26432245 10.1038/nature15393 PMC4750478

[43] Chhangawala S., Rudy G., Mason C.E., Rosenfeld J.A. The impact of read length on quantification of differentially expressed genes and splice junction detection. Genome Biol. 2015; 16:131. 26100517 10.1186/s13059-015-0697-y PMC4531809

