# Supplementary material for: CoolMPS for robust sequencing of single-nuclear RNAs captured by droplet-based method
Source: Nucleic Acids Res. 2020 Dec 2;49(2):e11. doi: 10.1093/nar/gkaa1127 (PMC7826285; doi:10.1093/nar/gkaa1127)
Supplement: gkaa1127_Supplemental_Files [file gkaa1127_supplemental_files.zip › OHahn 10-02-2020 Supplementary Figure legends.docx]

**Supplementary figure legends**

**Supplementary Figure 1. Distribution of reads derived from Chromium 10X chemistry over the 3’ end of all expressed genes**

(a) Aligned read density of aggregated reads from the converted libraries dataset over bins stretching 400 bp up- and down-stream of end of transcript (‘Eot’) of expressed genes, respectively.  (b) Knee plot of sample Y1 (young male replicate 1), showing UMI counts per cell barcode. Black points represent cells, with cells called only in one library type colored in red. These panels represent the highlighted areas in Figure 1e, marking the region at the cutoff between called cells and background (empty droplets).

**Supplementary Figure 2. Per-base quality scores before and after conversion**

(a-d,e-h) Per-base quality for (a-d) index read (read 1) and (e-h) fragment read (read 2), split by sample. First and last bases of forward strand were clipped. (i) % of reads with an average quality value of 30 (Q30) or higher. Abbreviations: Y1,Y2 - Young male replicates 1 and 2; O1,O2 - Old male replicates 1 and 2; R1, R2 - Read 1 (index read) and read 2 (sequenced fragment). (j) Distribution of the percentage of reads mapping to the mouse reference genome mm10 per technology, shown as boxplot and dotplot. Each sample is shown as one dot. The boxes span the first to the third quartile with the horizontal line inside the box representing the median value. The whiskers show the minimum and maximum values or values up to 1.5 times the interquartile range below or above the first or third quartile if outliers are present. (k) Distribution of the estimated error rate. Each sample is shown as one dot. The boxes span the first to the third quartile with the horizontal line inside the box representing the median value. The whiskers show the minimum and maximum values or values up to 1.5 times the interquartile range below or above the first or third quartile if outliers are present.

**Supplementary Figure 3. Read duplication values before and after conversion**

(a-d,e-h)  Distribution of the read duplication levels for the (a-d) index read (read 1) and (e-h) fragment read (read 2), split by sample and colored by library type.

**Supplementary Figure 4. % GC content before and after conversion**

(a-d,e-h)  Distribution of the per-read GC content for the (a-d) index read (read 1) and (e-h) fragment read (read 2), split by sample and colored by library type. (i) Distribution of the overall GC content for the index reads (read 1) and fragment reads (read 2). Each sample is shown as one dot. The boxes span the first to the third quartile with the horizontal line inside the box representing the median value. The whiskers show the minimum and maximum values or values up to 1.5 times the interquartile range below or above the first or third quartile if outliers are present.

**Supplementary Figure 5. Base-composition per cycle before and after conversion**

(a,b)  Average base composition of the index reads (read 1) and fragment reads (read 2), for the native (a) and converted (b) libraries. The gray area represents the standard deviation.

**Supplementary Figure 6. Contamination with mitochondrial DNA and ambient RNA are detected equally in native and converted libraries**

(a) % of UMI counts mapping to mitochondrial genes in native and CoolMPS-compatible dataset. (b) Overlap of cells from native and CoolMPS-compatible libraries excluded due to the following criteria : expression of fewer than 600 or more than 3500 genes; exhibiting mitochondrial read proportions higher than 0.5%. (c) Apparent and corrected expression levels of Ttr in sample O1 colored on tSNE, as detected in (c) the native dataset and (d) the CoolMPS-compatible dataset, respectively. (e) % of *SoupX*-predicted contamination of ambient RNA per sample. P-value = 0.12 for paired, two-sided Wilcoxon rank-sum test to probe for significant differences in contamination between native and converted libraries. (f,g) Scatterplot showing the cell-wise number of (f) detected UMIs and (g) genes by either library type after correction with SoupX. Insert shows the zoomed region with Deming regression line, showing a mild offset from the diagonal. Abbreviations: O1 - Old male replicate 1; Chor. Plx. - Choroid plexus cells; Mapping to mito. genome - mapping to mitochondrial genome; Est. contamination - estimated contamination; Ttr Expr. - scaled expression of gene *Ttr*. QC filtered cells - cells not passing the quality control filter; a.u. - arbitrary unit.
