# Supplementary figures and images for: CoolMPS for robust sequencing of single-nuclear RNAs captured by droplet-based method
Source: Nucleic Acids Res. 2020 Dec 2;49(2):e11. doi: 10.1093/nar/gkaa1127 (PMC7826285; doi:10.1093/nar/gkaa1127)

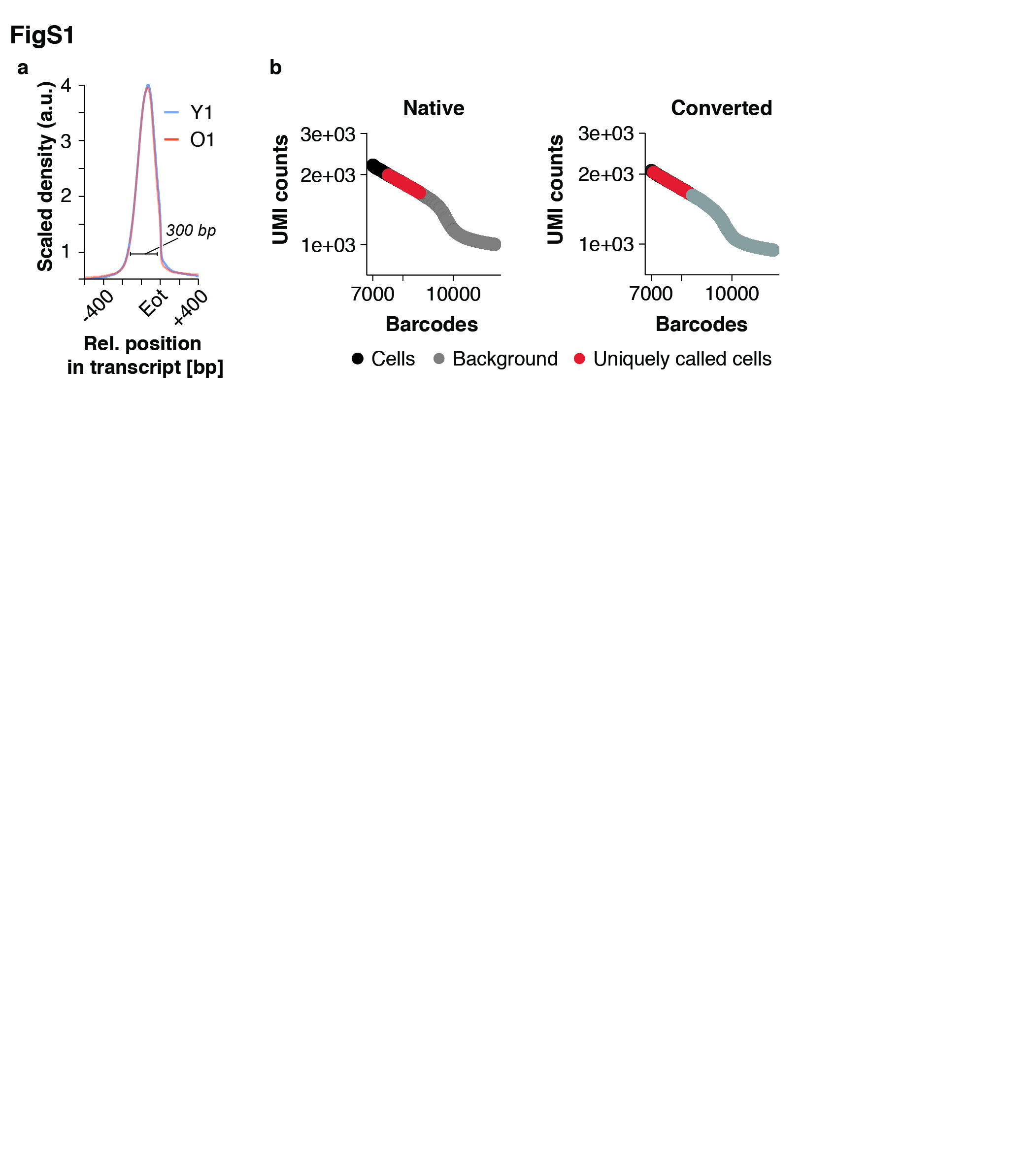

Supplement: gkaa1127_Supplemental_Files [file gkaa1127_supplemental_files.zip › _FigS1-01.tif]

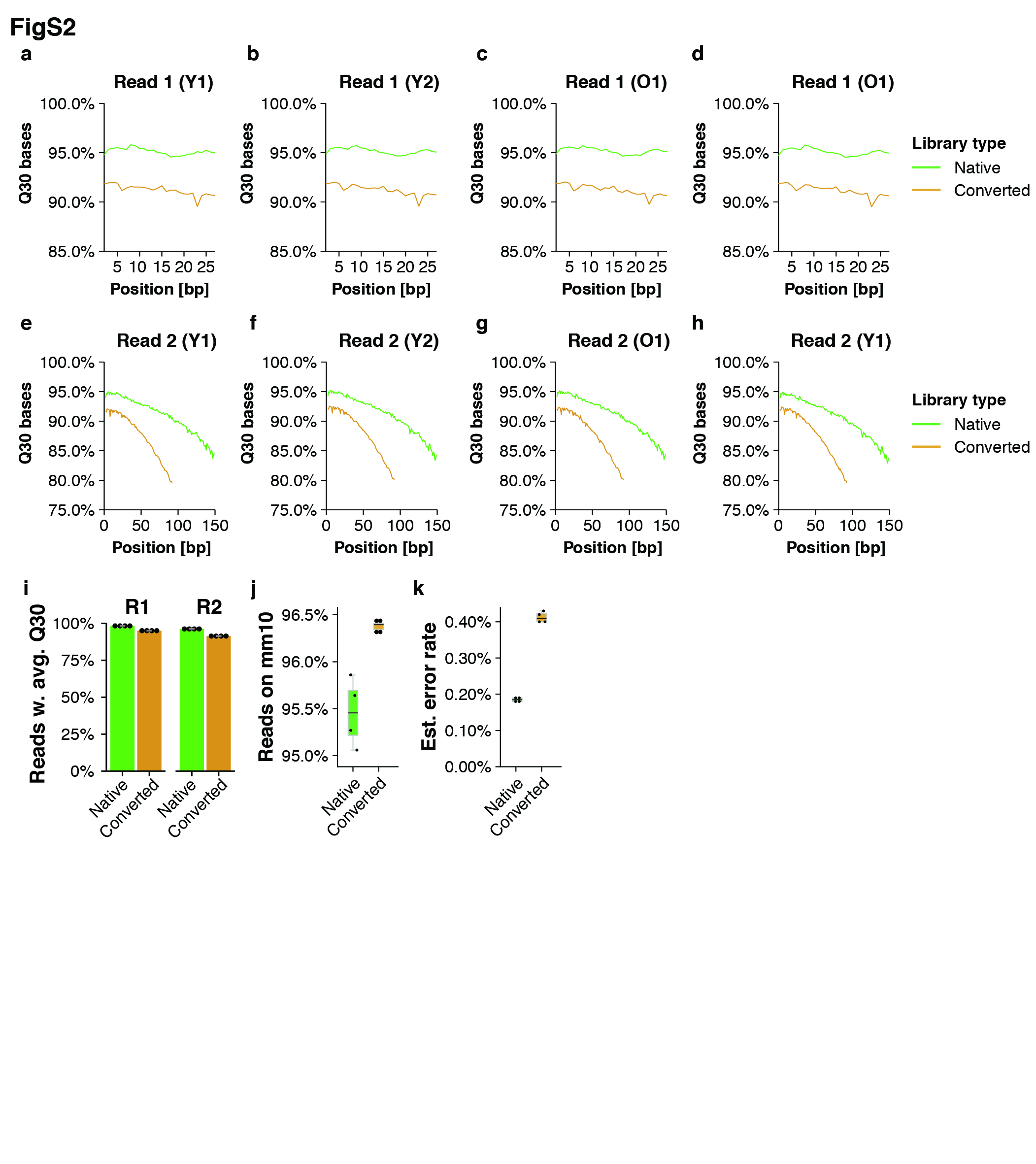

Supplement: gkaa1127_Supplemental_Files [file gkaa1127_supplemental_files.zip › _FigS2-01.tif]

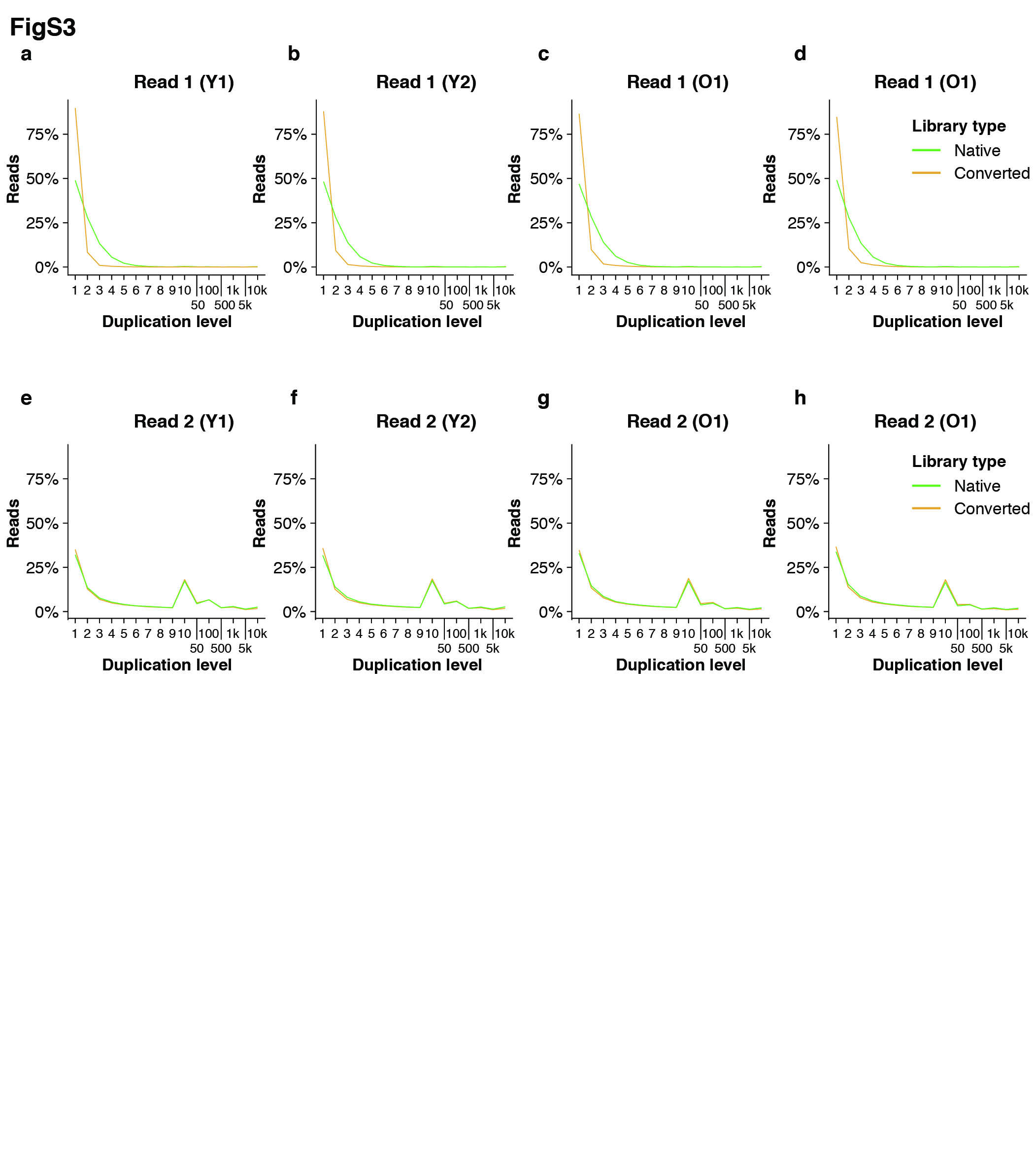

Supplement: gkaa1127_Supplemental_Files [file gkaa1127_supplemental_files.zip › _FigS3-01.tif]

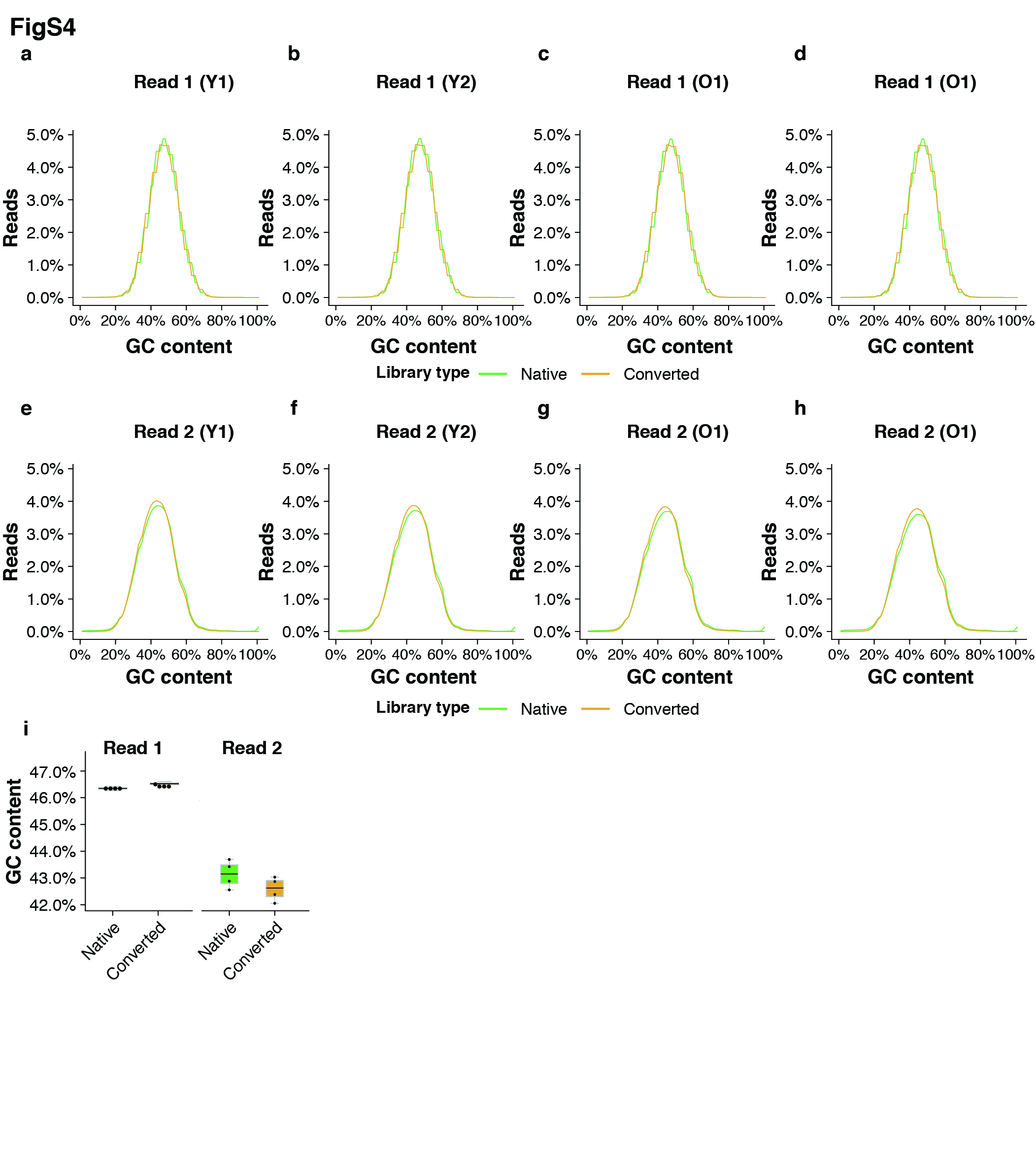

Supplement: gkaa1127_Supplemental_Files [file gkaa1127_supplemental_files.zip › _FigS4-01.tif]

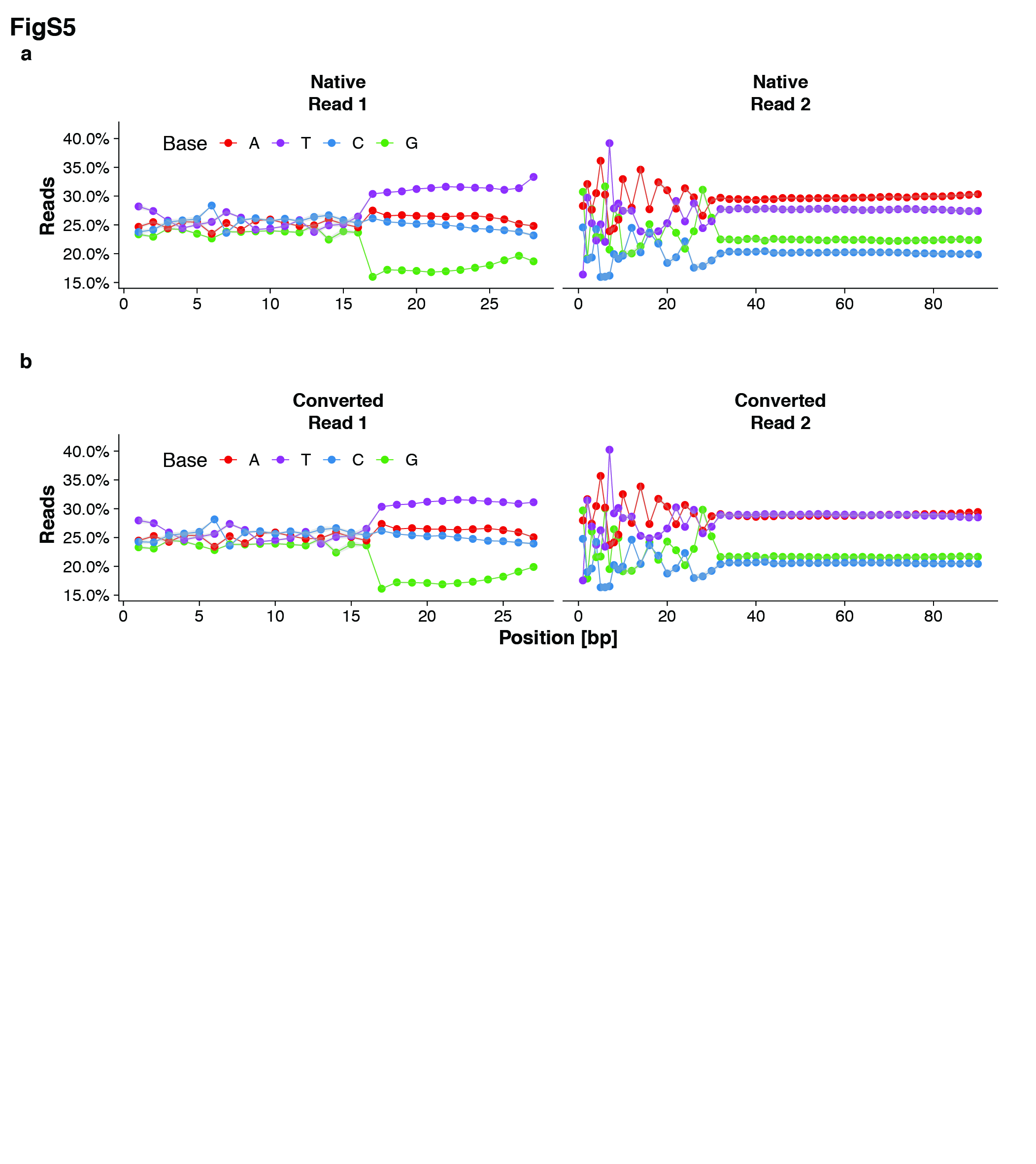

Supplement: gkaa1127_Supplemental_Files [file gkaa1127_supplemental_files.zip › _FigS5-01.tif]

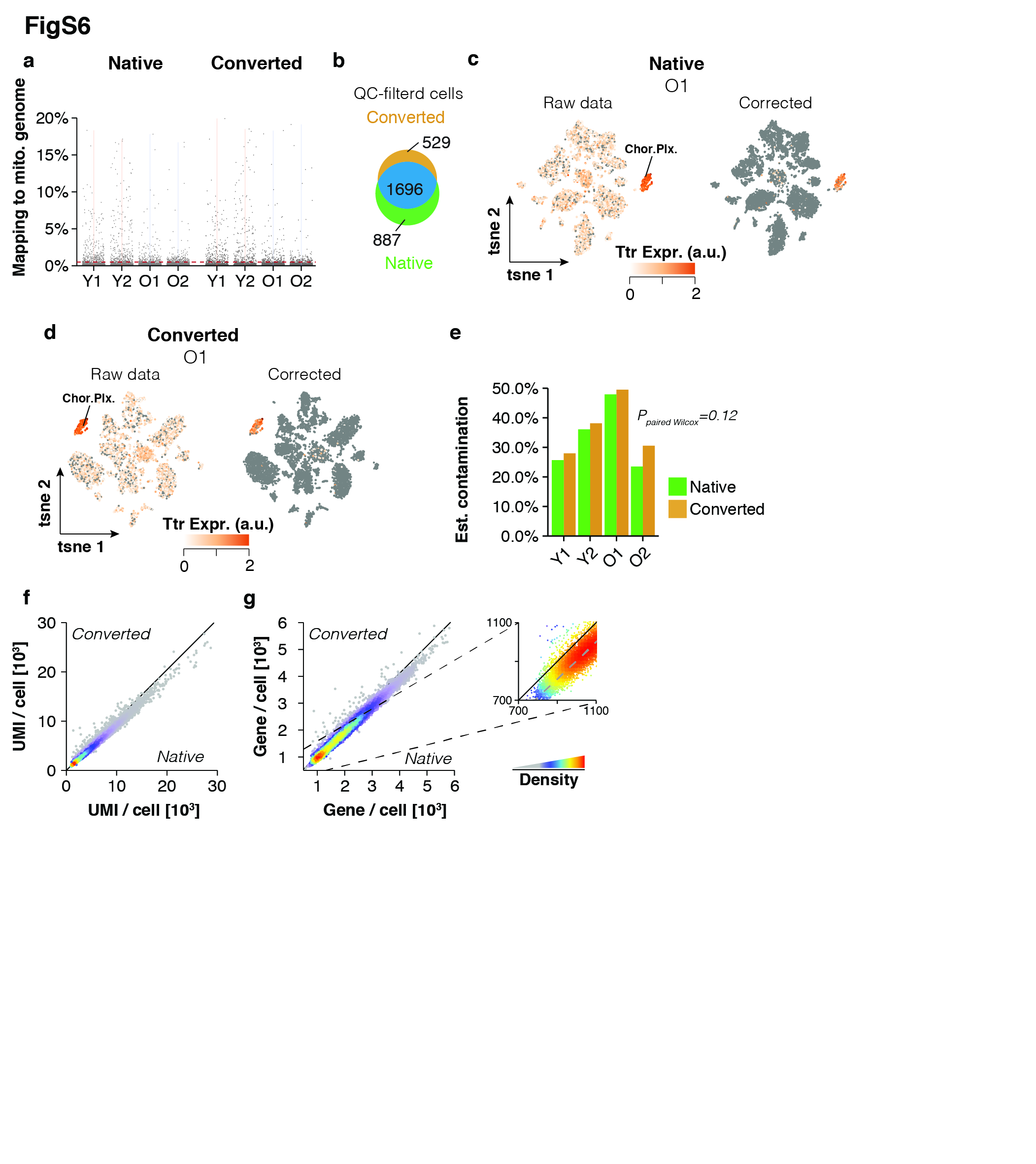

Supplement: gkaa1127_Supplemental_Files [file gkaa1127_supplemental_files.zip › _FigS6-01.tif]
